# Evaluating influential factors of acupuncture for tension-type headache: A protocol for systematic review and meta-analysis

**DOI:** 10.1097/MD.0000000000023118

**Published:** 2020-11-13

**Authors:** Xinyu Hao, Yang Shi, Jun Zhu, Jing Wang, Qianhua Zheng, Charlie Changli Xue, Ying Li, Zhen Zheng

**Affiliations:** a College of Acupuncture-Moxibustion and Tuina, Chengdu University of Traditional Chinese Medicine; b Acupuncture & Chronobiology Key Laboratory of Sichuan Province, Chengdu; c Chongqing University Cancer Hospital, Chongqing, China; d School of Health and Biomedical Sciences, RMIT University, Melbourne, Australia; e Graduate School, Chengdu University of Traditional Chinese Medicine, Chengdu, China.

**Keywords:** acupuncture, influential factors, meta-regression, systematic review, tension-type headache

## Abstract

**Background:**

Acupuncture has been recommended for the treatment of tension-type headache (TTH). However inconsistent findings on the efficacy of acupuncture impacts on the uptake of this effective therapy for TTH. This systematic review aims to 1) Evaluate the efficacy of acupuncture for TTH; and 2) Clarify the factors contributing to conflicting findings through conducting a meta-analysis and meta-regression of randomized controlled trials.

**Methods:**

Comprehensive literature search will be performed on PubMed, EMBASE, CINAHL, ProQuest, Cochrane Central Register of Controlled Trials, Acubriefs, ScienceDirect, Scopus, AMED, and 4 leading Chinese databases of the China National Knowledge Infrastructure, China Science and Technology Journal Database, Wanfang Data, and CBM (SinoMed). We will include Randomized controlled Trials or controlled trials of patients with TTH that compared acupuncture with sham interventions. The primary outcome is the number of days on headache (within 4 weeks) at the end of the treatment and follow-ups. Secondary outcomes include intensity of pain, frequency of attack, and the adverse effects resulting from the intervention. We will use pre-defined sub-group analysis and meta-regression to explore the influential factors of acupuncture effects. Heterogeneity assessment will be performed before carrying out meta-analysis, whereas the subgroup analyses and meta-regression will be used in verifying the possible factors of heterogeneity when significant heterogeneity detected.

**Results:**

Review Manager 5.3 software will be used for meta-analysis. The synthesis will be performed by generating forest plots. Meta-regression will be used to understand influential factors for acupuncture in patients with TTH.

**Conclusion:**

By utilizing techniques of meta-regression, this study will provide evidence toward to a more focused understanding of influential factors for acupuncture in patients with TTH. This systematic review will provide quality evidence for the optimization of acupuncture therapeutic regimen. It will facilitate the development of clinical practice guideline on acupuncture for TTH.

## 1 Introduction

Tension-type Headache (TTH) is found to be the most common type of primary headache and the second most prevalent chronic disorders.^[1]^ It is characteristically described as a dull, pressing, or tight quality without specific features in quality. The headache is mild to moderate in intensity, and the pain is usually bilateral, nonpulsatile, generally markedly persistent. It is a significant cause of distress and disruption to life, resulting in marked reductions in quality of life and engagement in social and family activities.^[3]^ However, the exact aetiology and pathophysiology of TTH remain unclear and the treatment strategies for TTH remain unspecific.^[2–4]^

Acupuncture, one of the most widely used complementary therapies has been reported to be effective for pain management.^[5]^ It is an invasive stimulation technique using thin needles on specific acupoints on the skin of the body. Based on Chinese medicine (CM) theory, it corrects imbalances in the flow of qi. One recent Cochrane review confirmed that acupuncture is effective for treating frequent episodic or chronic tension-type headaches.^[6]^ As a valuable non-pharmacological option for patients suffering from frequent episodic or chronic TTH,^[7]^ acupuncture has been recommended as a prophylactic treatment due to its effectiveness and safety profile.^[8]^ However, consensus on whether real acupuncture is superior to sham acupuncture has not been fully reached. Heterogeneity in trials might have contributed to the conflicting findings.

This systematic review is an update of our published study in 2013.^[9]^ In that study, we analyzed sources of heterogeneity and explored factors that have been considered to have direct impact on the quality of acupuncture trial. Those may include stimulation modes, needle retention time, and treatment frequency. However, the conclusion is limited due to a small number of trials. Among the 5 trials included, only 2 studies provided detailed information to enable us to compare the treatment protocol. In this proposed systematic review, we aim to evaluate the efficacy of acupuncture for TTH, and clarify the factors contributing to conflicting findings through conducting a meta-analysis and meta-regression of randomized controlled trials (RCTs) by comparing the effects of real and sham acupuncture on TTH. Specifically, this review will investigate the effect of major contributing factors that have been reported or considered to have direct impact on the quality of acupuncture trials.

## 2 Methods

### 2.1 Study registration

The protocol has been registered in the International Prospective Register of Systematic Reviews with registration number CRD42019134067.

### 2.2 Systematic review

The systematic review will follow the items of Preferred Reporting Items for Systematic Reviews and Meta-Analyses Protocol.^[10]^ The meta-analysis will be performed in accordance with the recommendations of Cochrane Handbook for Systematic Reviews of Interventions.^[11]^

### 2.3 Inclusion and exclusion criteria

#### 2.3.1 Types of studies

Included studies

(1) Are randomized or quasi-randomized controlled trials (RCTs or quasi-RCTs);
(2) Have adult patients;
(3) With TTH diagnosed according to International Headache Society criteria of the International Classification of Headache Disorders (ICHD-II, ICHD-III beta 2013, or ICHD-III)^[12–14]^ or the Ad Hoc committee on the Classification of Headache Ad Hoc 1962^[15]^;
(4) Reported headache days as an outcome measurement;
(5) Employed invasive acupuncture needling;
(6) Needled acupoints, Ashi point; and
(7) Compare against sham interventions:

Studies will be excluded if:

(1) Point injections are used because it is difficult to differentiate if the efficacy is from medication or acupuncture itself;
(2) Dry needling is employed because its theory and practice is not in accordance with traditional acupuncture; or
(3) Results about patients with TTH were not reported separately from those with other types of headache, such as migraine.

#### 2.3.2 Types of participants

Adult patients must have been diagnosed with TTH according to the International Headache Society diagnostic criteria^[12–14]^ or Ad hoc committee's criteria^[15]^ if studies conducted before 1988. Studies including patients with headaches of various types (e.g., patients have both tension-type headache and migraine) unless separate results are presented for patients with TTH will not be included.

#### 2.3.3 Types of intervention

In this review, acupuncture is limited to needle insertion type, that is , invasive methods, such as manual acupuncture, electro-acupuncture. Points needled include acupoints, Ashi point, trigger/tender points, and auricular acupoints. Interventions such as point injection, and dry-needling, laser acupuncture, acupoint catgut embedding will be excluded because its theory and practice is not in accordance with traditional acupuncture. Control intervention in this study is specified as sham control, acupuncture. Sham acupuncture refers to interventions mimicking “real” or “true” acupuncture treatment procedure, not in accordance with traditional acupuncture treatment process. Two types of conventional sham control are acceptable in this study, that is , using superficial needle insertion with real needles on non-acupuncture points, or, applying non-penetrating placebo acupuncture devices, such as the Park sham acupuncture device^[16]^ or the Streitberger needle.^[17]^

#### 2.3.4 Type of outcome measures

##### 2.3.4.1 Primary outcomes

Headache days (within 4 weeks)^[18]^ at the end of the treatment and follow-ups.

##### 2.3.4.2 Secondary outcomes

1) Intensity of pain (assessed by validated tool, e.g., visual analogue scale),
2) Frequency of attack (numbers of headache attacks per evaluation interval)
3) Adverse effects resulting from the intervention

### 2.4 Search methods for identification of studies

#### 2.4.1 Data source

Thirteen major databases will be searched, consisting of 9 English databases (PubMed, EMBASE, CINAHL, ProQuest, Cochrane Central Register of Controlled Trials, Acubriefs, ScienceDirect, Scopus, AMED) and 4 leading Chinese databases (Chinese databases of China National Knowledge Infrastructure, China Science and Technology Journal Database, Wanfang Data and CBM (SinoMed)) from the inception to December of 2019. An updated search will be performed prior to the data analysis.

#### 2.4.2 Search strategy

As shown in Table 1, MeSH terms and keywords in combination for search are tension-type headache, acupuncture, randomized controlled trial and their variations. There will be no restrictions on language of publication.

**Table 1:** Search strategy and search terms.

A. Search strategy to locate “tension-type headache”
# 1. Tension-type headache (MeSH)
# 2. Tension headache (tw)
# 3. Headache (MeSH)
# 4. TTH (tw)
# 5. TH (tw)
# 6. or/# 1-# 5
B. Search strategy to locate acupuncture interventions
# 7. Acupuncture (MeSH)
# 8. Acupuncture therapy (MeSH)
# 9. Electroacupuncture (MeSH)
# 10. Electro-acupuncture (tw)
# 11. Brief needling (tw)
# 12. Dry needling (tw)
# 13. Electrical acupuncture (tw)
# 14. Acupuncture points (MeSH)
#15. Body acupuncture (tw)
# 16. Scalp acupuncture (tw)
# 17. Routine acupuncture (tw)
# 18. Manual acupuncture (tw)
# 19. Abdomen acupuncture (tw)
# 20. Or/#7–#19
C. Search strategy to locate RCTs or semi RCT
# 21. Randomized controlled trial (MeSH)
# 22. RCT (tw)
# 23. Controlled trial (tw)
# 24. CT (tw)
# 25. or/#21-#24
D. Search strategy to locate studies for this review
#6 and #20 and #25

### 2.5 Data collection and analysis

#### 2.5.1 Selection of studies

**Figure 1:**
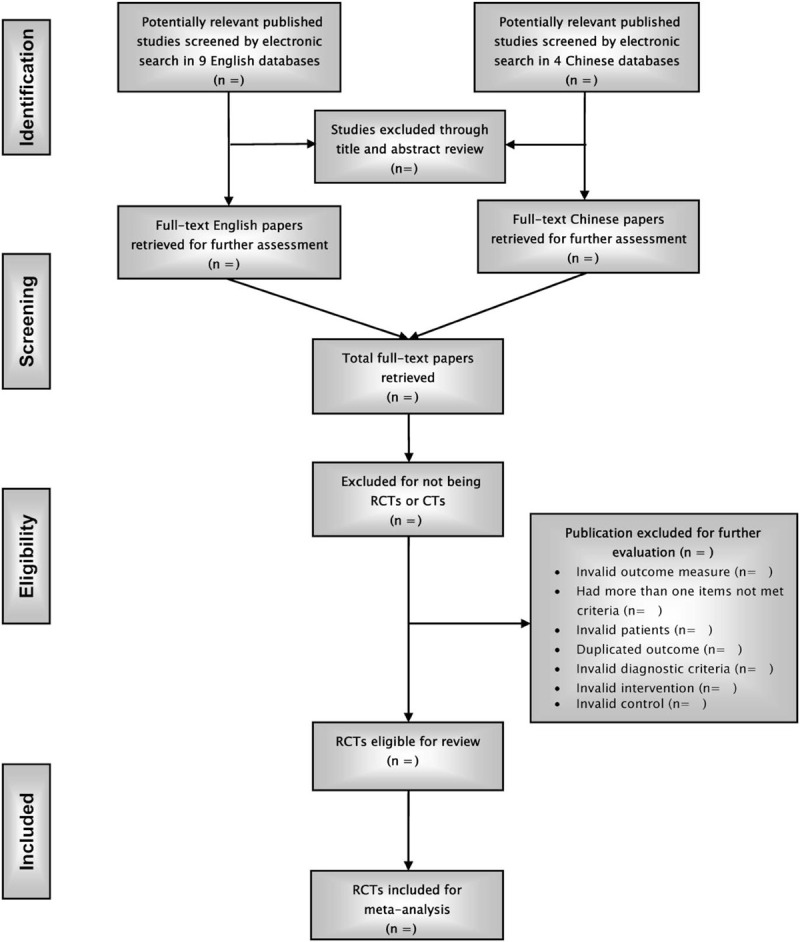
Flow diagram of study selection process.

The screening process will use EndNote X9 literature management software (Clarivate Analytics US LLC, Philadelphia) for management and deduplication. Two reviewers will read the titles and abstracts to exclude irrelevant trials. Full text will be obtained for the determination of the potential eligible studies against the inclusion and exclusion criteria. The entire screening processes will be performed independently and in duplicate. Disagreements will be solved by discussion and consult with a third reviewer. Authors will be contacted for insufficient information and missing data. Reasons for the excluded studies will be recorded, and the selection process will be presented in the Preferred Reporting Items for Systematic Reviews and Meta-Analyses flow diagram (Fig. 1).

#### 2.5.2 Data extraction

The 2 reviewers will extract data from eligible trials independently by using a standard pre-defined data extraction form, which covers the following components:

1) Study characteristics: authors, year of publication, study design, sample size, interventions and controls, methods randomisation blinding, and allocation concealment;
2) Outcome data: mean and standard deviation/standard error will be extracted for continuous outcomes, whereas the number of events and total population will be extracted for dichotomous outcomes;
3) Patients characteristics: age (mean age) or age range, sex (male, %), ethnic population, numbers of randomized treated, analyzed and their withdrawals of data, and primary and secondary outcomes at all reported time points;
4) Acupuncture treatment characteristics: number of needles, total number of treatments, frequency of treatments, timing of treatments, point selected, needle retention time, mode of stimulation, achievement of De-qi, manipulation technique on needles, rational on point selection, feature of sham control stimulation styles, and repot of background or training information of practitioners, which are in line with the Standards for Reporting Interventions in Controlled Trials of Acupuncture criteria.^[19]^

Other information including protocols of treatment and selection criteria will be also extracted for study comparisons.

#### 2.5.3 Risk of bias assessment

For the assessment of study quality, the Gordon H. Guyatt's revision of Cochrane risk of bias tool for the assessment^[11,20]^ will be used, which involves 7 specific domains of

1) Random sequence generation,
2) Allocation concealment,
3) Blinding of patients and caregivers,
4) Blinding of outcome assessment,
5) Blinding of patients and caregivers,
6) Selective outcome reporting, and
7) Other bias.

Methodological quality will be considered as “low risk” of bias, “high risk” of bias or “unclear risk” of bias.

#### 2.5.4 Assessment of reporting bias

Egger test^[21]^ and funnel plots will be used to assess the publication bias if there are at least 10 trials included in meta-analysis. A *P* < .05 indicates a significant publication bias.

### 2.6 Data synthesis

RevMan 5.3 (Review Manager (RevMan) [Computer program]. Version 5.3. Copenhagen: The Nordic Cochrane Centre, The Cochrane Collaboration, 2014) software provided by Cochrane collaboration (www.cochrane.org) will be used for meta-analysis. The synthesis will be performed by generating a forest plot for meta-regression. A random-effect model will be used if significant heterogeneity (I^2^≥50%) among the trials is detected. For continuous data, weight or standard mean difference will be used. When different scales are used for assessing 1 outcome measure, standard mean difference will be calculated.

#### 2.6.1 Assessment of heterogeneity

Heterogeneity of the included studies will be evaluated by using the Cochrane Q test and it will be quantified with I^2^ statistics. A low, moderate and high I^2^ value will be indicated by 25%, 50%, and 75% respectively.^[22]^

#### 2.6.2 Subgroup analysis and meta-regression

Subgroup analyses and meta-regression will be used in exploring the possible factors of heterogeneity. Subgroup analysis will be performed to verify the sources of heterogeneity contributed to the results if a significant heterogeneity was detected.^[11]^ Based on our previous findings of potential and influential factors,^[9]^ the following variables are predefined for analysis:

1) Type of acupuncture (manual acupuncture vs electrical acupuncture)
2) Needle retention (needle retention vs no-retention)
3) Frequency of treatment (twice a week or more vs once a week)
4) Point selection (semi-standardized vs formula treatment)
5) Number for study center (multicenter vs single site)
6) CM diagnosis guided treatment (trial treatments are based on CM diagnosis and vs treatments are standardized without considering CM diagnosis).
7) Design of sham acupuncture (having sham points on the head vs not on the head).

The post-hoc subgroup analyses and meta-regression for other independent variables will also be considered subject to other necessary variables.

#### 2.6.3 Sensitivity analysis

Sensitivity analysis will be performed to evaluate the robustness of the pooled results, by excluding studies with “high risk” and “unclear risk” of bias.

#### 2.6.4 Quality of evidence assessment

The quality of evidence will be assessed using the Grading of Recommendations Assessment, Development, and Evaluation approach^[23]^ the Grading of Recommendations Assessment, Development, and Evaluation will be used as a tool for assessing the strength of the body of evidence. In this evaluation system, the strength of evidence can be divided into 4 levels as “high”, “moderate”, “low”, or “very low” by the outcome.

### 2.7 Ethics and dissemination

The results of this meta-analysis and meta-regression will be disseminated in a peer reviewed journal. Ethics approval is not required in this study as no individualized data will be involved.

## 3 Discussion

Several systematic reviews discussed effectiveness and efficacy of acupuncture for TTH, including a 2008 systematic review,^[24]^ 1 Cochrane review published in 2009^[6]^ and an update version on 2016.^[9]^ In contrast to the published reviews, our previously review^[9]^ use subgroup analyses to explore the contributing factors to the inconsistent findings. Due to a relatively small number of eligible clinical trials and limited evidence, we were not able to provide conclusive recommendations on the contributing factors.

In this proposed study, we will include more trials and use meta-regression. Those strategies will provide quality evidence towards a more focused understanding of influential factors for acupuncture in patients with TTH. Our findings will facilitate the development of clinical practice guideline of acupuncture for TTH and contribute to the optimized acupuncture treatment regimen for the enhanced therapeutic effects.

## Author contributions

Xinyu Hao, Yang Shi, Ying Li, and Zhen Zheng conceived the systematic review. Xinyu Hao and Yang Shi contributed equally as first co-authors and wrote the final draft. All the authors helped to write the article and approved the publication of the protocol.

**Conceptualization:** Xinyu Hao, Yang Shi, Ying Li, Zhen Zheng.

**Data curation:** Xinyu Hao, Jun Zhu, Jing Wang, Qianhua Zheng.

**Formal analysis:** Xinyu Hao. Yang Shi.

**Methodology:** Xinyu Hao, Charlie Changli Xue.

**Supervision:** Zhen Zheng, Ying Li.

**Writing – original draft:** Xinyu Hao, Yang Shi.

**Writing – review & editing:** Xinyu Hao, Yang Shi.

## References

[1] Vos T Barber R Bell B Global, regional, and national incidence, prevalence, and years lived with disability for 301 acute and chronic diseases and injuries in 188 countries, 1990-2013: a systematic analysis for the global burden of disease study 2013. Lancet 2015;386:743–800. 26063472 10.1016/S0140-6736(15)60692-4 PMC4561509

[2] Devlin I Selvaratnam P Niere K Zuluaga M Headache in general practice. Churchill Livingstone, Headache, Orofacial Pain and Bruxism. Edinburgh: 2009.

[3] Mueller L Tension-type, the forgotten headache. How to recognize this common but undertreated condition. Postgrad Med 2002;111:25–6. 10.3810/pgm.2002.04.1165 11985132

[4] Jensen R Diagnosis epidemiology and impact of tension-type headache. Curr Pain Headache Rep 2003;7:455–9. 14604504 10.1007/s11916-003-0061-x

[5] Ezzo J Berman B Hadhazy VA Is acupuncture effective for the treatment of chronic pain? A systematic review. Pain 2000;86:217–25. 10812251 10.1016/S0304-3959(99)00304-8

[6] Linde K Allais G Brinkhaus B Acupuncture for tension-type headache. Cochrane Database Syst Rev 2009;CD007587. 19160338 10.1002/14651858.CD007587 PMC3099266

[7] Carville S Padhi S Reason T Diagnosis and management of headaches in young people and adults: summary of NICE guidance. BMJ 2012;345:e5765. 22993393 10.1136/bmj.e5765

[8] Linde K Allais G Brinkhaus B Acupuncture for the prevention of tension-type headache. Cochrane Database Syst Rev 2016;4:CD007587. 27092807 10.1002/14651858.CD007587.pub2 PMC4955729

[9] Hao XA Xue CC Dong L Factors associated with conflicting findings on acupuncture for tension-type headache: qualitative and quantitative analyses. J Altern Complement Med 2013;19:285–97. 23075410 10.1089/acm.2011.0914

[10] Moher D Shamseer L Clarke M Preferred reporting items for systematic review and meta-analysis protocols (PRISMA-P) 2015 statement. Syst Rev 2015;4:1. 25554246 10.1186/2046-4053-4-1 PMC4320440

[11] In: Higgins JPT, Green S, eds. Cochrane Handbook for Systematic Reviews of Interventions Version 5.2.0. 2017: The Cochrane Collaboration. www.cochrane-handbook.org. Accessed 20 May 2020.

[12] Headache Classification Subcommittee of the International Headache Society. The International Classification of Headache Disorders: 2nd edition. Cephalalgia 2004;24: Suppl 1: 9–160. 14979299 10.1111/j.1468-2982.2003.00824.x

[13] Headache Classification Committee of the International Headache Society (IHS). The International Classification of Headache Disorders, 3rd edition (beta version). Cephalalgia 2013;33:629–808. 23771276 10.1177/0333102413485658

[14] Headache Classification Committee of the International Headache Society (IHS). The international classification of headache disorders. 3rd edition. Cephalalgia 2018;38:1–211. 10.1177/0333102417738202 29368949

[15] Nappi G Agnoli A Manzoni GC Classification and diagnostic criteria for primary headache disorders (Ad Hoc committee IHS, 1988). Funct Neurol 1989;4:65–71. 2737496

[16] Park J White A Lee H Development of new sham needle. Acupunct Med 1999;17:110–2.

[17] Streitberger K Kleinhenz J Introducing a placebo needle into acupuncture research. Lancet 1998;352:364–5. 9717924 10.1016/S0140-6736(97)10471-8

[18] Bendtsen L Bigal ME Cerbo R Guidelines for controlled trials of drugs in tension-type headache: second edition. Cephalalgia 2010;30:1–6. 19614696 10.1111/j.1468-2982.2009.01948.x

[19] MacPherson H Altman DG Hammerschlag R Revised standards for reporting interventions in clinical trials of acupuncture (STRICTA): extending the CONSORT statement. J Altern Complement Med 2010;16:ST1–4. 20954957 10.1089/acm.2010.1610

[20] Guyatt GH Busse JW Tool to assess risk of bias in randomized controlled trials 2017. Available at: https://www.evidencepartners. com/wp-content/uploads/2017/09/Tool-to-Assess-Risk-of-Bias-in-Ran domized-Controlled-Trials.pdf. Accessed March 18, 2020.

[21] Higgins JP Thompson SG Deeks JJ Measuring inconsistency in meta-analyses. BMJ 2003;327:557–60. 12958120 10.1136/bmj.327.7414.557 PMC192859

[22] Egger M Davey Smith G Schneider M Bias in meta-analysis detected by a simple, graphical test. BMJ 1997;315:629–34. 9310563 10.1136/bmj.315.7109.629 PMC2127453

[23] Balshem H Helfand M Schünemann HJ GRADE guidelines: 3. rating the quality of evidence. J Clin Epidemiol 2011;64:401–6. 21208779 10.1016/j.jclinepi.2010.07.015

[24] Davis MA Kononowech RW Rolin SA Acupuncture for tension-type headache: a meta-analysis of randomized, controlled trials. J Pain 2008;9:667–77. 18499526 10.1016/j.jpain.2008.03.011

